# Beta Interferon Production Is Regulated by p38 Mitogen-Activated Protein Kinase in Macrophages via both MSK1/2- and Tristetraprolin-Dependent Pathways

**DOI:** 10.1128/MCB.00454-16

**Published:** 2016-12-19

**Authors:** Victoria A. McGuire, Dalya Rosner, Olga Ananieva, Ewan A. Ross, Suzanne E. Elcombe, Shaista Naqvi, Mirjam M. W. van den Bosch, Claire E. Monk, Tamara Ruiz-Zorrilla Diez, Andrew R. Clark, J. Simon C. Arthur

**Affiliations:** a MRC Protein Phosphorylation Unit, School of Life Sciences, Sir James Black Centre, University of Dundee, Dundee, United Kingdom; b Division of Cell Signaling and Immunology, School of Life Sciences, Sir James Black Centre, University of Dundee, Dundee, United Kingdom; c Institute of Inflammation and Ageing, College of Medical and Dental Sciences, University of Birmingham, Birmingham, United Kingdom

**Keywords:** CREB, DUSP1, beta interferon, MSK1, MSK2, TTP, p38 kinases

## Abstract

Autocrine or paracrine signaling by beta interferon (IFN-β) is essential for many of the responses of macrophages to pathogen-associated molecular patterns. This feedback loop contributes to pathological responses to infectious agents and is therefore tightly regulated. We demonstrate here that macrophage expression of IFN-β is negatively regulated by mitogen- and stress-activated kinases 1 and 2 (MSK1/2). Lipopolysaccharide (LPS)-induced expression of IFN-β was elevated in both MSK1/2 knockout mice and macrophages. Although MSK1 and -2 promote the expression of the anti-inflammatory cytokine interleukin 10, it did not strongly contribute to the ability of MSKs to regulate IFN-β expression. Instead, MSK1 and -2 inhibit IFN-β expression via the induction of dual-specificity phosphatase 1 (DUSP1), which dephosphorylates and inactivates the mitogen-activated protein kinases p38 and Jun N-terminal protein kinase (JNK). Prolonged LPS-induced activation of p38 and JNK, phosphorylation of downstream transcription factors, and overexpression of IFN-β mRNA and protein were similar in MSK1/2 and DUSP1 knockout macrophages. Two distinct mechanisms were implicated in the overexpression of IFN-β: first, JNK-mediated activation of c-jun, which binds to the IFN-β promoter, and second, p38-mediated inactivation of the mRNA-destabilizing factor tristetraprolin, which we show is able to target the IFN-β mRNA.

## INTRODUCTION

The production of cytokines by macrophages is an important event in both the initiation and coordination of immune responses. Macrophages detect invading pathogens via a series of germ line-encoded pattern recognition receptors (PRRs), such as Toll-like receptors (TLRs); NOD-like receptors (NLRs); CARD helicases, such as RIG-I; and C-type lectin receptors, including dectin-1 (1–3). PRRs are able to detect specific classes of pathogen-associated molecular patterns (PAMPs), and the binding of PAMPs to their respective PRRs activates the intracellular signaling pathways required to promote the appropriate cellular responses, including the production of cytokines, to mount an effective immune response. Following activation of PRRs, macrophages produce a range of proinflammatory cytokines, including tumor necrosis factor (TNF), interleukin 1 (IL-1), IL-6, and IL-12. Significantly, the precise profile of cytokines produced is tailored to the specific PRR, or combination of PRRs, that is activated. Thus, the response of the macrophage can be optimized for the type of invading pathogen (1–3).

TLRs are the best studied of the different PRRs in macrophages and consist of a family of 10 receptors in humans and 12 in mice (1–4). Toll-like receptors act either at the plasma membrane or in endosomes and couple with downstream signaling via a family of four adaptor proteins and can therefore be classified according to their primary sites of action and the specific adaptors they require. With the exception of TLR3, all TLRs are able to activate signaling via the MyD88 adaptor (1). For TLR2 and TLR4, a further adaptor, Mal, is required for efficient recruitment of MyD88 to the receptor. Via MyD88, TLRs are able to activate the canonical NF-κB signaling pathway, as well as the extracellular signal-regulated kinase 1/2 (ERK1/2), p38, and Jun N-terminal protein kinase (JNK) mitogen-activated protein kinase (MAPK) cascades (5, 6). Together, these pathways act to induce transcription of the cytokines required to promote an immune response. In contrast to other TLRs, TLR3 signals via Trif and not MyD88. While TLR3 can activate NF-κB and MAPK signaling, it also induces the activation of TBK1 and IκB kinase ε (IKKε), which results in the phosphorylation and activation of the transcription factor interferon regulatory factor 3 (IRF3) (7). This promotes the transcription of the type I interferon beta interferon (IFN-β). TLR4 is unusual in that, in addition to signaling via MyD88, it can also interact with Trif, whose recruitment is promoted by a further adaptor, TRAM. The ability to interact with Trif means that TLR4, unlike other MyD88-dependent TLRs, can also activate IRF3 and efficiently stimulate IFN-β production by macrophages (8). The importance of IFN-β in mediating the effects of TLR4 activation has been shown by the observation that IFN-β knockout mice are protected from lipopolysaccharide (LPS)-induced endotoxic shock (9) while an IFN-β feedback loop is required for the induction of multiple genes in response to LPS (8, 10, 11).

While inflammation is an important process for dealing with infection, unchecked inflammation has serious adverse consequences. A number of negative-feedback pathways and anti-inflammatory mediators have therefore evolved to prevent this from occurring. In addition to the production of proinflammatory cytokines, macrophages also produce the anti-inflammatory cytokines IL-10 and IL-1 receptor antagonist (IL-1ra) in response to PRRs (12–15). Once secreted, IL-10 restimulates macrophages and represses the induction of proinflammatory cytokines via a STAT3-dependent mechanism (16), while IL-1ra acts as a competitive inhibitor of IL-1 signaling (13). These represent important feedback mechanisms that serve to prevent excessive inflammation and tissue damage. The effects of loss-of-function mutations in humans illustrate the importance of these cytokines to maintaining balance in the immune system. Inactivating mutations in IL-10 or the IL-10 receptor promote severe early-onset colitis (17, 18). This parallels the mouse phenotype, as IL-10 knockout mice are sensitive to the spontaneous development of colitis (19).

In response to TLR4, IL-10 transcription is regulated by the ERK1/2 and p38 MAPK pathways, and an important role for the kinases mitogen- and stress-activated kinase 1 (MSK1) and MSK2 in this process has been identified (20–22). MSK1 and -2 are activated in cells via phosphorylation by either ERK1/2 or p38α and can therefore serve to integrate signals downstream of these two pathways (23). The major role of MSKs appears to be in the regulation of specific genes downstream of MAPK activation (21, 22, 24), a function that is reflected in the known substrates of MSKs. For example, MSKs can phosphorylate the chromatin proteins histone H3 and HMG14, as well as the transcription factors CREB and ATF1 (25–28). Mice with double knockout of both MSK1 and -2 are viable and do not exhibit any adverse welfare effects under standard conditions (28). However, more detailed analysis has revealed phenotypes in these mice, particularly with respect to the central nervous system (CNS) and innate immune system (21, 22, 29, 30). MSK1/2 knockout in mice results in sensitization to LPS-induced endotoxic shock, which correlates with increased plasma TNF and IL-12 levels but decreased IL-10 and IL-1ra levels (21, 31). More detailed studies in macrophages have indicated that MSKs can directly regulate IL-10 transcription, and the reduction in IL-10 secretion is a major driving force behind the observed increases in proinflammatory cytokines in MSK1/2 knockout mice (31). The predominant mechanism by which MSKs regulate IL-10 in response to LPS is via the phosphorylation of CREB (21). Binding sites for CREB have been identified in the IL-10 promoter, and mutation of the MSK phosphorylation site, Ser133, in CREB reduced TLR4-induced IL-10 transcription (21, 32–34).

In addition to its regulation by MSK1/2 downstream of TLR activation, the production of IL-10 in response to LPS stimulation can be sustained in macrophages via an IFN-β-mediated feedback loop (35). While a role for MSKs in the regulation of IL-10 has been established, the effect of MSK activation on type I interferon production has not been addressed. We show here that knockout of MSK1 and -2 in macrophages results in increased production of IFN-β via increased activation of the p38 and JNK signaling pathways.

## RESULTS

### MSK1/2 inhibit LPS-induced IFN-β *in vivo* and in isolated macrophages.

**FIG 1:**
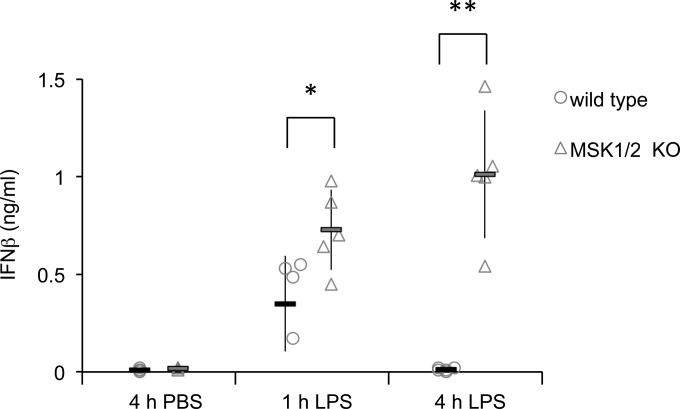
MSK1/2 regulate LPS-induced IFN-β production *in vivo*. Wild-type or MSK1/2 knockout mice were given an intraperitoneal injection of 1.8 mg/kg LPS in PBS or a control injection of PBS. Sera were collected at 1 or 4 h postinjection, and IFN-β levels were measured by ELISA. The results for individual mice are shown by symbols, while averages are shown by bars. The error bars represent the standard deviations of 4 or 5 mice per condition. KO, knockout. *, *P* < 0.05; **, *P* < 0.01 (two-tailed Student *t* test).

To examine the role of MSK1 and -2 in regulating IFN-β production, mice were given an intraperitoneal (i.p.) injection of LPS, and the plasma IFN-β levels were determined 1 or 4 h later. In wild-type mice, IFN-β was strongly induced after 1 h, but this increase was not sustained at 4 h. In contrast, MSK1/2 knockout mice produced higher and more sustained induction of plasma IFN-β levels (Fig. 1).

**FIG 2:**
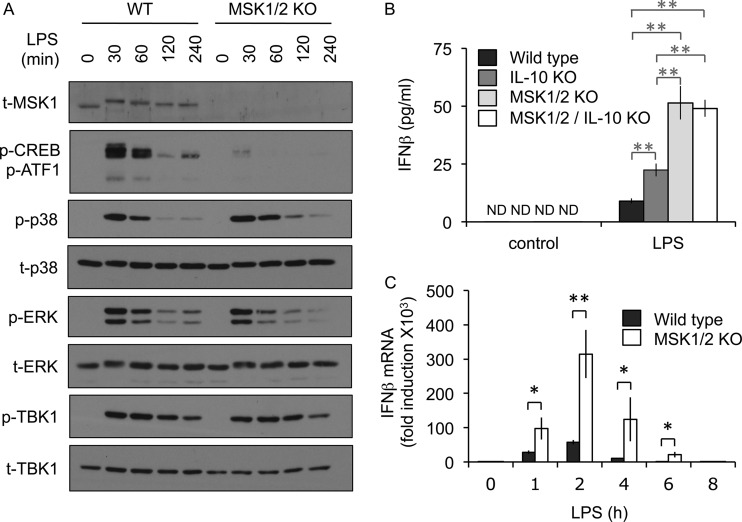
MSKs inhibit IFN-β production downstream of TLR4. (A) Wild-type (WT) or MSK1/2 knockout BMDMs were stimulated with 100 ng/ml LPS for the indicated times. The cells were then lysed, and the levels of total MSK1 (t-MSK1), phospho-CREB (p-CREB)/p-ATF1, phospho- and total p38, phospho- and total ERK1/2, and phospho- and total TBK1 were determined by immunoblotting. (B) BMDMs were prepared from wild-type, IL-10 knockout, MSK1/2 double-knockout, or MSK1/2 IL-10 triple-knockout animals. The cells were stimulated with 100 ng/ml LPS for 8 h, and the levels of IFN-β secreted into the medium were determined by ELISA. The error bars represent the standard deviations of independent cultures from 3 mice per genotype. ND, not detected. (C) Wild-type or MSK1/2 knockout BMDMs were stimulated with 100 ng/ml LPS for the indicated times, and IFN-β mRNA levels relative to the wild-type unstimulated cells were determined by qPCR. The error bars represent the standard deviations of independent cultures from 4 mice per genotype. *, *P* < 0.05; **, *P* < 0.01 (two-tailed Student *t* test).

As MSK1/2 knockout mice produced more IFN-β in response to LPS, we examined the induction of IFN-β in wild-type and MSK1/2 knockout bone marrow-derived macrophages (BMDMs). As has previously been reported (21), MSKs were activated in BMDMs in response to LPS, as judged by a band shift in the total MSK1 blots and the phosphorylation of the MSK1/2 substrates CREB and ATF1 (Fig. 2A). Double knockout of MSK1 and -2 prevented the phosphorylation of CREB. MSK1/2 knockout did not block the phosphorylation of ERK1/2 and p38α in response to LPS, although, as has been reported previously (21), prolonged activation of p38α was observed in the MSK1/2 knockout cells. IFN-β production in response to LPS requires the activation of the TBK1/IRF3 pathway; however, MSK1/2 knockout did not increase the phosphorylation of TBK1 on sites that correspond to its activation in response to LPS relative to that seen in wild-type BMDMs (Fig. 2A). In line with the *in vivo* data on IFN-β, MSK1/2 knockout macrophages produced elevated levels of IFN-β in response to LPS compared to wild-type cells (Fig. 2B). One possible explanation for this could be the decreased level of IL-10 production that has previously been described in MSK1/2 knockout BMDMs (21). To address this, we examined the effect of MSK1/2 knockout on IFN-β production in an IL-10-deficient background. IL-10 knockout BMDMs produced more IFN-β in response to LPS than wild-type cells; however, the increase was not as great as that seen between wild-type and MSK1/2 knockout BMDMs (Fig. 2B). Comparison of the IL-10–MSK1/2 triple knockouts with MSK1/2 knockouts showed that loss of IL-10 in the absence of MSK1/2 did not affect IFN-β production (Fig. 2B). Together, these data indicate that MSK1/2 regulate IFN-β production, at least in part, via a mechanism independent of IL-10. As MSKs regulated IFN-β production, we next determined if this might be due to a change in IFN-β mRNA induction. LPS was found to induce IFN-β mRNA in both wild-type and MSK1/2 knockout BMDMs; however, knockout of MSK1 and -2 resulted in induction of IFN-β mRNA higher than that seen in wild-type cells (Fig. 2C).

**FIG 3:**
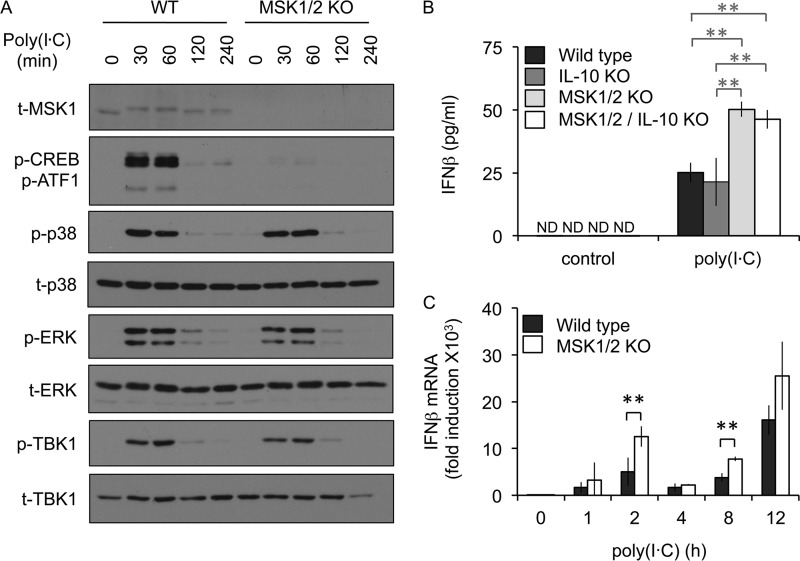
MSKs inhibit IFN-β production downstream of TLR3. (A) Wild-type or MSK1/2 knockout BMDMs were stimulated with 10 μg/ml poly(I·C) for the indicated times. The cells were then lysed, and the levels of total MSK1, phospho-CREB/ATF1, phospho- and total p38, phospho- and total ERK1/2, and phospho- and total TBK1 were determined by immunoblotting. (B) BMDMs were prepared from wild-type, IL-10 knockout, MSK1/2 double-knockout, or MSK1/2 IL-10 triple-knockout animals. The cells were stimulated with 10 μg/ml poly(I·C) for 8 h, and the levels of IFN-β secreted into the medium were determined by ELISA. The error bars represent the standard deviations of independent cultures from 3 mice per genotype; ND, not detected. (C) Wild-type or MSK1/2 knockout BMDMs were stimulated with 10 μg/ml poly(I·C) for the indicated times, and IFN-β mRNA levels relative to the wild-type unstimulated cells were determined by qPCR. The error bars represent the standard deviations of independent cultures from 4 mice per genotype. **, *P* < 0.01 (two-tailed Student *t* test).

In addition to TLR4 agonists such as LPS, TLR3 stimulation also induces IFN-β production in macrophages (36). As for LPS, poly(I·C) induced the activation of MSK1 and the phosphorylation of the MSK1/2 substrates CREB and ATF1 (Fig. 3A). MSK1/2 knockout BMDMs produced more IFN-β than wild-type cells in response to poly(I·C) (Fig. 3B). In contrast to the results for LPS, single IL-10 knockout did not affect the levels of IFN-β mRNA production in response to poly(I·C). This may reflect the much higher levels of IL-10 produced in response to LPS than to poly(I·C) in wild-type cells (362 pg/ml versus 25 pg/ml in this experiment). In line with the data on IFN-β secretion, MSK1/2 knockout also resulted in increased induction of IFN-β mRNA in response to poly(I·C) (Fig. 3C). The effects of MSK1/2 knockout on poly(I·C)-induced IFN-β production, however, were less pronounced than those seen with LPS (compare Fig. 2 and 3). Together, the above-mentioned results indicate that an IL-10- and TBK1-independent mechanism exists that allows MSK activation to inhibit IFN-β production in macrophages.

### MSK modulates JNK activation in response to LPS.

**FIG 4:**
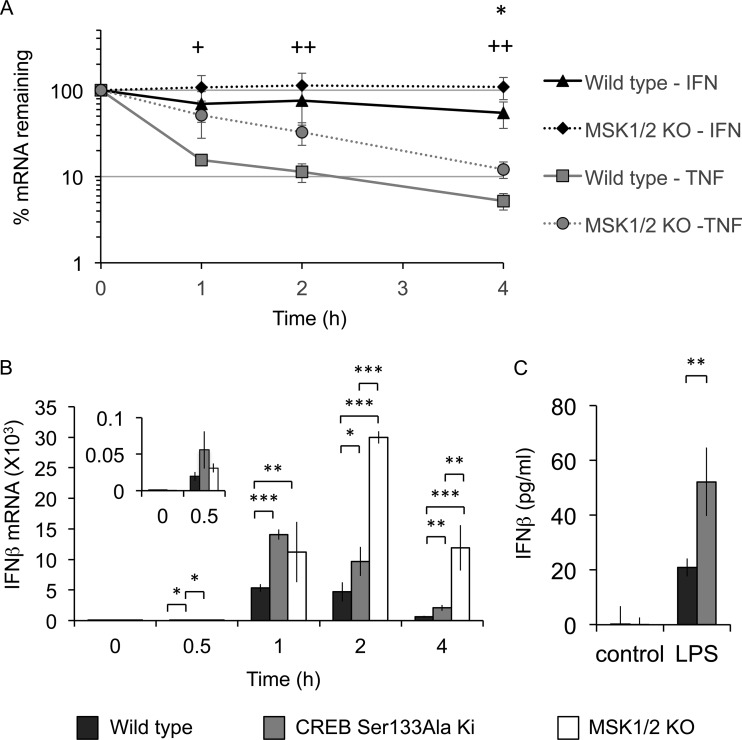
MSK-dependent effects on IFN-β mRNA induction are mediated through CREB. (A) Wild-type or MSK1/2 knockout BMDMs were stimulated with 100 ng/ml LPS for 2 h, and then 1 μg/ml actinomycin D was added. At the indicated times after actinomycin D addition, the cells were lysed, and TNF and IFN-β mRNA levels were determined by qPCR. The results are expressed as the percent mRNA level relative to the zero time point and were normalized against the average of the 18S and GAPDH RNA levels. The error bars represent the standard deviations of 4 independent cultures per genotype. For IFN-β, *, *P* < 0.05 (two-tailed Student *t* test) for comparisons of wild type and MSK1/2 knockout. For TNF, +, *P* < 0.05, and ++, *P* < 0.01 between wild type and knockout. (B) Wild-type, MSK1/2 knockout, or CREB Ser133Ala knock-in BMDMs were stimulated with 100 ng/ml LPS for the indicated times, and IFN-β mRNA levels were determined by qPCR. (C) Wild-type or CREB Ser133Ala knock-in BMDMs were stimulated with 100 ng/ml LPS for 8 h, and the levels of IFN-β secreted into the medium were determined by ELISA. (B and C) The error bars represent the standard deviations of independent cultures from 4 mice per genotype. *, *P* < 0.05; **, *P* < 0.01; ***, *P* < 0.001 (two-tailed Student *t* test).

Changes in mRNA levels can reflect a change in transcription or mRNA stability (37). Analysis of primary transcript levels can provide an indication of transcription rates; however, the method depends on detection of unspliced pre-mRNA, which is not possible for IFN-β, as it contains no introns. To look at mRNA stability, cells were stimulated with LPS for 2 h, and then actinomycin D was used to block transcription. Analysis of TNF mRNA showed that it was rapidly degraded following actinomycin D treatment (Fig. 4A), a finding consistent with previous studies. Knockout of MSK1 and -2 resulted in stabilization of TNF mRNA (Fig. 4A), consistent with the previous finding that MSKs regulated TNF in part via an IL-10-independent mechanism (21). IFN-β mRNA was more stable than TNF mRNA in these experiments (Fig. 4A). However, IFN-β mRNA did follow a trend toward more stability in the MSK1/2 knockout cells than in wild-type cells, but it reached statistical significance only at the 4-h time point (Fig. 4A). The relative stability of the IFN-β mRNA at 2 h of LPS stimulation suggests that changes in IFN-β transcription also play a role in the ability of MSKs to control IFN-β transcription at early time points following LPS stimulation.

MSKs are able to regulate transcription via the phosphorylation of CREB. We therefore examined the effect of a knock-in mutation of the MSK phosphorylation site in CREB, Ser133, on IFN-β mRNA induction. Macrophages from CREB Ser133Ala knock-in mice showed increased induction of IFN-β mRNA relative to wild-type cells following LPS stimulation (Fig. 4B), although the effect was not as pronounced as that seen in the MSK1/2 knockout cells. This could reflect compensation from the related transcription factor ATF1 in the CREB knock-in cells or a role for another MSK1/2 substrate. In line with the increased IFN-β mRNA in the CREB Ser133Ala cells, LPS-induced IFN-β secretion was also increased in the CREB Ser133Ala knock-in relative to wild-type BMDMs (Fig. 4C).

While IFN-β mRNA induction was increased in CREB Ser133Ala knock-in BMDMs, a CREB site has not previously been found in the IFN-β promoter. It is possible that the effects of MSK and CREB are not direct and occur via an MSK-CREB target gene.

**FIG 5:**
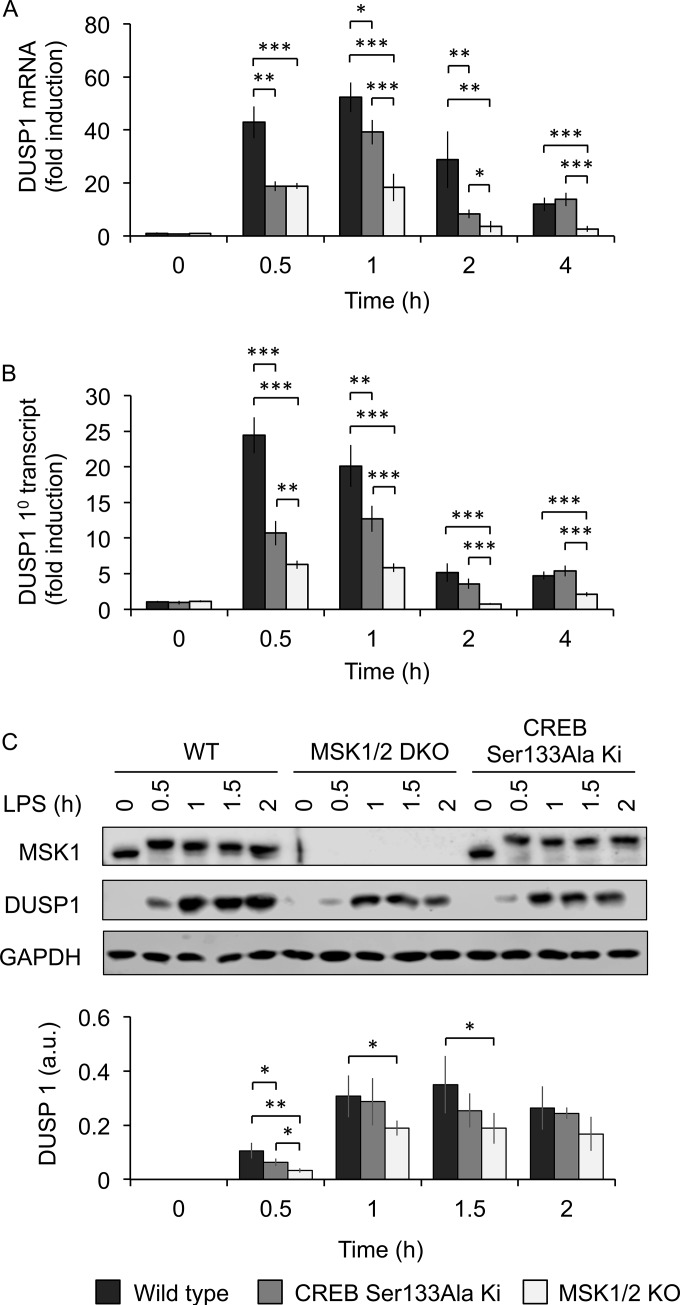
MSK regulates DUSP1 induction. Wild-type, MSK1/2 knockout, or CREB Ser133Ala knock-in BMDMs were stimulated for the indicated times with 100 ng/ml LPS. (A and B) The cells were then lysed, and the levels of DUSP1 mRNA (A) or DUSP1 primary transcript (B) were determined by qPCR. (C) Alternatively, cells were stimulated for the indicated times and lysed, and the levels of total MSK1, DUSP1, and GAPDH were determined by immunoblotting. The signals for DUSP1 from the immunoblots were quantified and corrected for the average of GAPDH and total JNK levels (a representative blot for JNK is shown in Fig. 6). The graphs show means, and the error bars represent the standard deviations of independent cultures from 4 (A and B) or 3 (C) mice per genotype. *, *P* < 0.05; **, *P* < 0.01; ***, *P* < 0.001 (two-tailed Student *t* test).

In addition to IRF3, a role for an AP-1 complex made up of c-jun and ATF2 on the IFN-β promoter has been described (38, 39). Furthermore, small interfering RNA (siRNA) against c-jun has been shown to decrease TLR4-stimulated induction of IFN-β in macrophages (40). c-jun activity can be controlled by phosphorylation by JNK. We therefore examined if MSKs might indirectly regulate IFN-β induction via cross talk with the JNK pathway. MSK1/2 and CREB have been linked to the transcription of dual-specificity phosphatase 1 (DUSP1) (21, 41), a phosphatase that can dephosphorylate both JNK and p38 in macrophages (42–45). We therefore investigated if MSKs might regulate LPS-stimulated JNK activity, and therefore c-jun phosphorylation, via DUSP1 in BMDMs. In agreement with previous studies, MSK1/2 knockout reduced the induction of DUSP1 mRNA in response to LPS (Fig. 5A). As MSKs phosphorylate CREB on Ser133 and as DUSP1 is a CREB target gene (21, 41), we examined if DUSP1 transcription was decreased in macrophages with a Ser133Ala knock-in mutation in the CREB gene. LPS-induced DUSP1 induction was reduced by the CREB Ser133Ala knock-in, although not to the same extent as for MSK1/2 knockout (Fig. 5A). To examine if this change was likely to be due to a change in DUSP1 transcription, primary transcript levels were analyzed. This showed that either MSK1/2 knockout or CREB Ser133Ala knock-in reduced the induction of the primary DUSP1 transcript (Fig. 5B). In line with the RNA data, DUSP1 protein induction in response to LPS was also reduced (Fig. 5C), consistent with previous reports (21).

**FIG 6:**
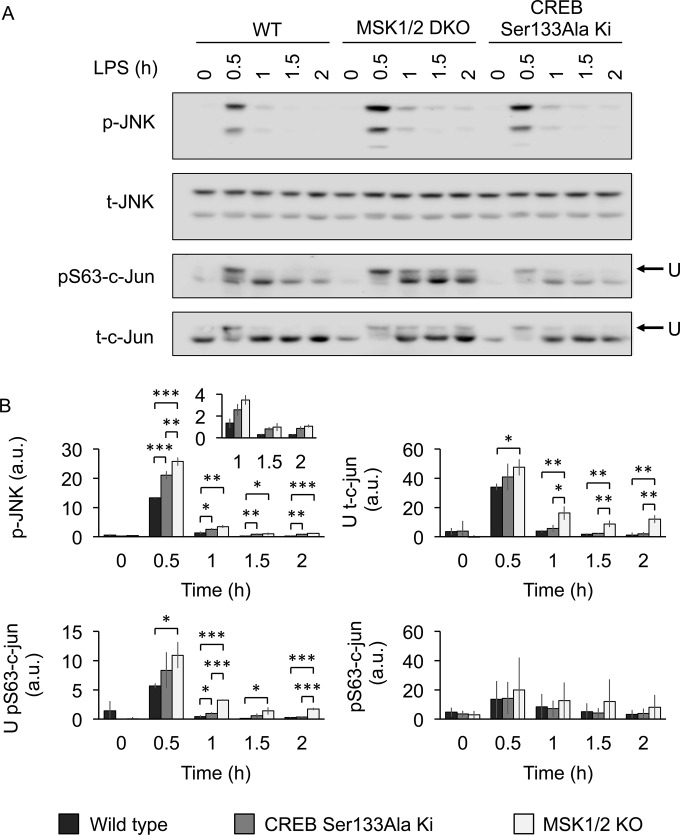
Regulation of JNK and c-jun phosphorylation by MSKs downstream of LPS. (A) Wild-type, MSK1/2 knockout, or CREB Ser133Ala knock-in BMDMs were stimulated with 100 ng/ml LPS for the indicated times. The cells were then lysed, and the levels of total and phosphoproteins shown were determined by immunoblotting. The blots are a representative example of independent cultures from 3 mice per genotype. (B) Signals from panel A were quantified, and the graphs show the levels of p-JNK corrected for total JNK, the upper c-jun band (U) corrected for the total c-jun signal, pS63 c-jun levels corrected for total c-jun, and the upper band in the pS63 blots corrected for total c-jun. The error bars represent the standard deviations of 3 independent cultures per genotype. a.u., arbitrary units. *, *P* < 0.05; **, *P* < 0.01; ***, P < 0.001 (two-tailed Student *t* test).

DUSP1 knockout has previously been shown to result in prolonged phosphorylation of JNK in macrophages (42–45). In agreement with the reduced induction of DUSP1 mRNA, there was prolonged phosphorylation of JNK on its TXY activation motif in response to LPS in MSK1/2 knockout relative to wild-type BMDMs (Fig. 6A). A similar but less pronounced effect was seen in the CREB Ser133Ala knock-in macrophages. To confirm these results, JNK phosphorylation was measured in biological replicates and quantified using a LiCor imager (Fig. 6B). The magnitude of this increase in the MSK1/2 knockout cells was similar to that observed in another DUSP1 substrate, p38α (Fig. 2A) (21) and is in line with what has been published for DUSP1 knockout (42–45). As JNK activation was more prolonged in the MSK1/2 knockout cells, the phosphorylation of c-jun was also examined. JNK has been reported to phosphorylate multiple sites in these proteins, and their phosphorylation in cells results in a bandshift of the protein on immunoblots (46–48). LPS stimulation resulted in a bandshift in blots using a total c-jun antibody that was maximal 30 min after stimulation. This bandshift was more prolonged in cells lacking MSK1 and -2 (Fig. 6). Again, an intermediate response was observed in the CREB Ser133Ala knock-in cells (Fig. 6). c-jun is phosphorylated on multiple sites, including Ser63. Immunoblotting with a Ser63 c-jun phosphospecific antibody showed that phosphorylation of Ser63 was induced by LPS. However, the effect of MSK1/2 knockout on the intensity of the bands detected with the phospho-Ser63 antibody was minor, suggesting that Ser63 was not the major site in c-jun affected by MSK1/2 knockout.

### DUSP1 inhibits IFN-β induction in response to LPS.

**FIG 7:**
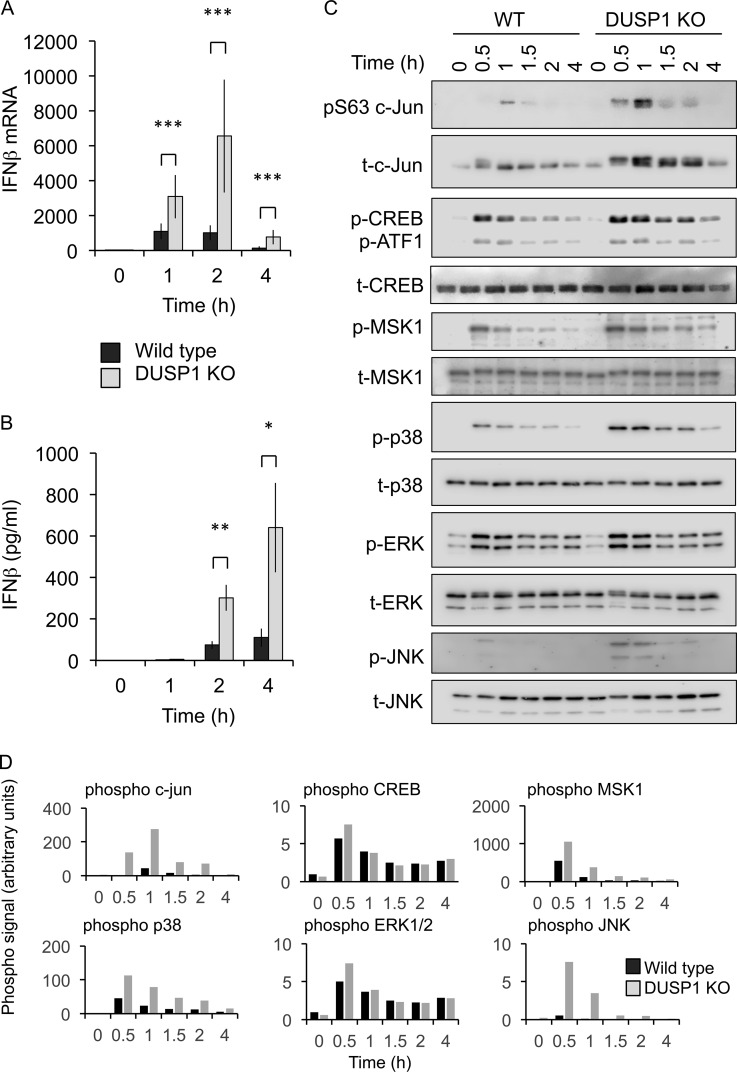
Knockout of DUSP1 increases IFN-β mRNA induction in response to LPS. (A) Wild-type or DUSP1 knockout BMDMs were stimulated with 100 ng/ml LPS for the indicated times. IFN-β mRNA levels were then determined by qPCR. The error bars represent the standard deviations of independent cultures from 12 mice per genotype. (B) Wild-type or DUSP1 knockout BMDMs were stimulated with 100 ng/ml LPS for the indicated times. IFN-β secretion levels were then determined by a Luminex-based assay as described in Materials and Methods. The error bars represent the standard deviations of independent cultures from 3 mice per genotype. (C) Wild-type or DUSP1 knockout BMDMs were stimulated with 100 ng/ml LPS for the indicated times and analyzed by immunoblotting for the total and phosphoproteins shown. (D) Phosphorylated bands in panel C were quantified, and the signal was corrected for protein loading based on the total-protein blots. *, *P* < 0.05; **, *P* < 0.01; ***, P < 0.001 (two-tailed Student *t* test).

If MSKs regulate IFN-β induction via a DUSP1-JNK-dependent mechanism, then DUSP1 knockout should phenocopy the MSK1/2 knockout with regard to IFN-β transcription. To examine this, DUSP1 knockout BMDMs were analyzed. Similar to MSK1/2 knockouts, DUSP1 knockouts showed higher induction of IFN-β mRNA in response to LPS than wild-type BMDMs (Fig. 7A). This was reflected in IFN-β secretion, as DUSP1 knockouts produced larger amounts of IFN-β than wild-type cells in response to LPS (Fig. 7B). In line with previous reports, loss of DUSP1 resulted in prolonged p38α and JNK activation following LPS stimulation (Fig. 7C and D). Of note, the effect on p38 phosphorylation was more pronounced in the DUSP1 knockout than in the MSK1/2 knockout (Fig. 2), which may be because MSK1/2 knockout reduces but does not abolish DUSP1 induction. DUSP1 also resulted in prolonged phosphorylation of c-jun, as judged by the bandshift in total c-jun blots (Fig. 7C). Interestingly DUSP1 knockout also resulted in prolonged MSK1 and CREB phosphorylation (Fig. 7C). This is most likely explained by the prolonged activation of p38α in the absence of DUSP1, resulting in MSK1/2 activation.

**FIG 8:**
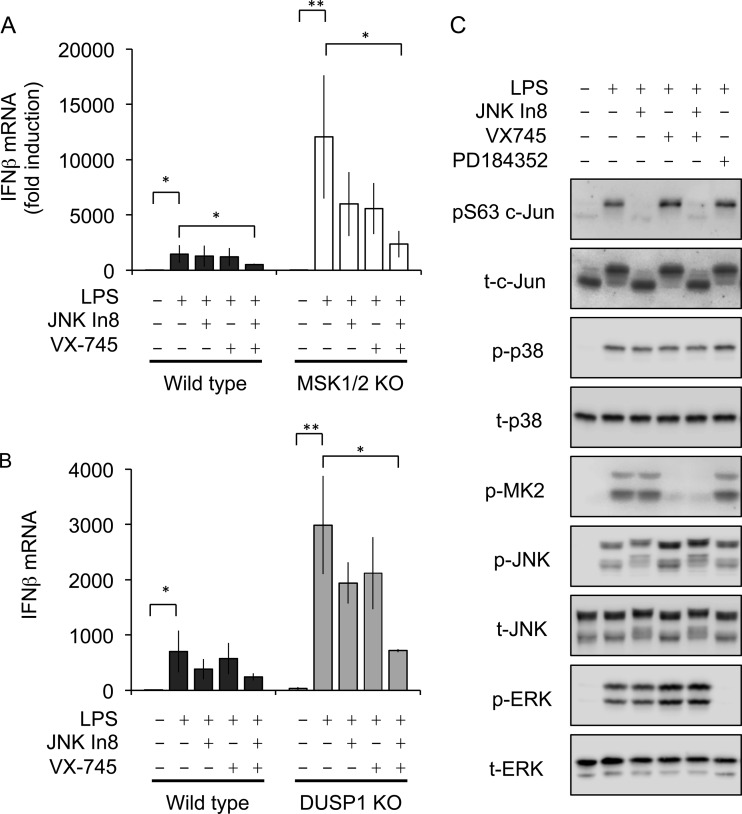
Effects of MAPK pathway inhibitors on c-Jun phosphorylation and IFN-β mRNA induction. (A) Wild-type or MSK1/2 knockout BMDMs were preincubated with1 μM VX745 for 1 h or with 3 μM JNK-In-8 for 3 h as indicated. The cells were then stimulated with 100 ng/ml LPS for 2 h, and IFN-β mRNA levels were determined by qPCR. The error bars represent the standard deviations of independent cultures from 4 mice per genotype. *, *P* < 0.05; **, *P* < 0.01 (two-tailed Student *t* test). (B) Wild-type or DUSP1 knockout BMDMs were preincubated with 1 μM VX745 for 1 h or 3 μM JNK-In-8 for 3 h as indicated. The cells were then stimulated with 100 ng/ml LPS for 2 h, and IFN-β mRNA levels were determined by qPCR. The error bars represent the standard deviations of independent cultures from 3 mice per genotype. *P* values between the wild type and DUSP1 knockout: *, *P* < 0.05; **, *P* < 0.01 (two-tailed Student *t* test). (C) Wild-type BMDMs were preincubated with 2 μM PD184352 or 1 μM VX745 for 1 h or 3 μM JNK-In-8 for 3 h as shown. The cells were then stimulated with 100 ng/ml LPS for 30 min, and the levels of the indicated total and phosphoproteins were determined by immunoblotting.

If MSK1/2 and DUSP1 repress IFN-β mRNA induction via inhibiting JNK and c-jun activation, inhibitors of JNK should reduce IFN-β mRNA levels in MSK1/2 or DUSP1 knockout cells. To test this, cells were preincubated either with the JNK inhibitor JNK-In-8 or the p38 inhibitor VX745 before stimulation with LPS. In MSK1/2 knockouts, JNK-In-8 resulted in a partial reduction of IFN-β mRNA induction, although IFN-β mRNA was not reduced to the levels observed in wild-type cells (Fig. 8A). Interestingly, VX745 also reduced IFN-β mRNA induction in MSK1/2 knockout BMDMs, while a combination of JNK-In-8 and VX745 suppressed IFN-β mRNA to a greater extent than either inhibitor alone (Fig. 8A). Similar results were also obtained for p38 and JNK inhibition in DUSP1 knockout BMDMs (Fig. 8B).

While JNK has been reported to be the major kinase for c-jun phosphorylation, roles for other MAPKs, including p38, have been proposed (46). To confirm that JNK was the predominant MAPK isoform for JNK phosphorylation in LPS-stimulated BMDMs, selective inhibitors of the ERK1/2 (PD184352), p38 (VX745), and JNK (JNK-In-8) pathways were used (Fig. 8C). None of the inhibitors tested affected p38 phosphorylation following 30 min of LPS stimulation. Inhibition of p38 blocked the phosphorylation of its substrate, MK2. p38 inhibition also resulted in an increase in LPS-stimulated JNK and ERK1/2 phosphorylation. This may reflect a previously described role for p38 in the feedback inhibition of the upstream kinase Tak1 or the induction of DUSP1 (20, 49). Neither p38 nor ERK1/2 inhibition affected c-jun phosphorylation (Fig. 8C). JNK-In-8 is a covalent inhibitor of JNK, and binding of the compound to JNK results in a bandshift of JNK on gels (50). Inhibition of JNK blocked c-jun phosphorylation as measured either by the bandshift in the total c-jun blots or with the Ser63 phosphospecific antibody (Fig. 8C).

### IFN-β mRNA stability is regulated by TTP.

**FIG 9:**
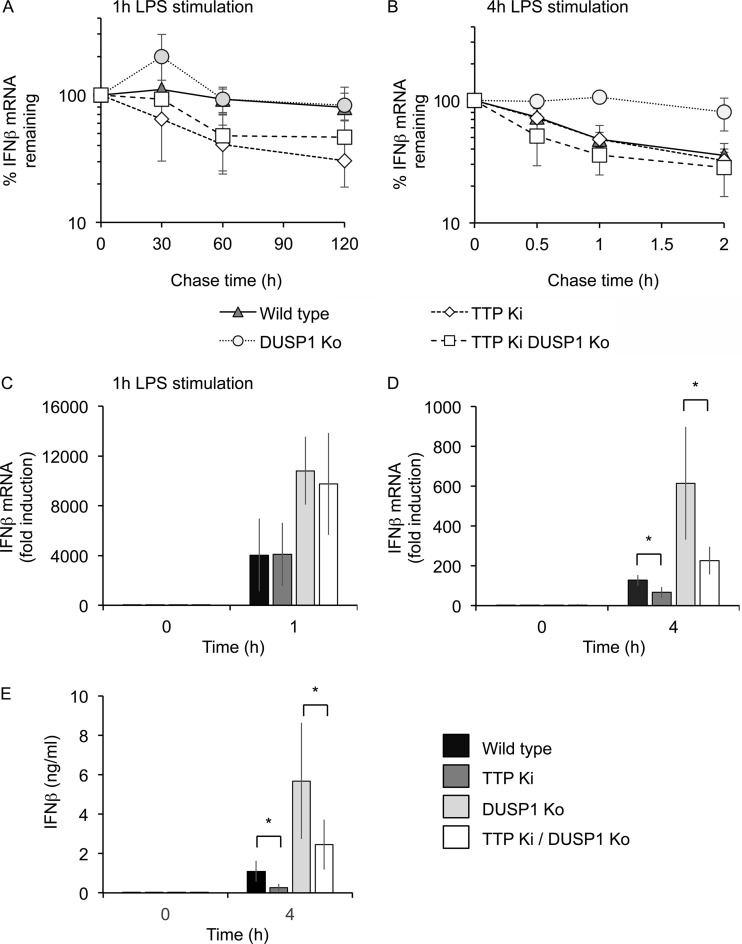
TTP regulates IFN-β production in response to LPS. (A and B) BMDMs were isolated from wild-type, DUSP1 knockout, TTP knock-in, or DUSP1/TTP double-mutant mice. The cells were stimulated with 100 ng/ml LPS for either 1 h (A) or 4 h (B), and then 50 μM 5,6-dichloro-1β-1-ribofuranosylbenzimidazole (DRB), and 5 μg/ml actinomycin D was added. IFN-β mRNA levels were then determined at the indicated times after DRB/actinomycin D addition. The levels were calculated relative to the LPS-stimulated cells prior to DRB/actinomycin D addition. The results represent the means and standard deviations of independent cultures from 3 mice per genotype. (C and D) BMDMs were isolated from wild-type, DUSP1 knockout, TTP knock-in, or DUSP1/TTP double-mutant mice. The cells were stimulated with 100 ng/ml LPS, and the levels of IFN-β mRNA were determined at 0, 1, and 4 h. Due to the different fold inductions, the results are shown on separate graphs for 1 h (C) and 4 h (D). The results represent individual cultures from 3 wild-type mice or 4 mice for other genotypes. The error bars represent standard deviations. (E) As for panel D, but the levels of IFN-β protein secreted into the medium were determined by a Luminex-based assay. The results represent the means and standard deviations of independent cultures from 4 mice per genotype. (C to E) For comparisons of TTP knock-in to wild type or DUSP1/TTP double mutants to DUSP1 knockout, *, *P* < 0.05 (two-tailed Student *t* test).

The finding that p38 inhibition reduced IFN-β induction in both MSK1/2 and DUSP1 knockouts but did not prevent c-jun phosphorylation indicates that p38 has additional roles in regulating IFN-β induction. p38 has previously been found to regulate the stability of mRNAs containing AU-rich elements (AREs) via the ARE binding protein TTP (51–57). At 2 h post-LPS stimulation, IFN-β mRNA was relatively stable in actinomycin D chase experiments (Fig. 4A). However, the transient nature of the IFN-β mRNA induction implies that IFN-β mRNA becomes less stable during prolonged LPS stimulation, and in line with this, IFN-β mRNA was less stable in actinomycin D chase experiments performed after 4 h of LPS stimulation compared to 1 h of LPS stimulation (compare Fig. 9A and B).

p38 activates the kinase MK2, which in turn can phosphorylate TTP at serines 52 and 178, preventing it from recruiting deadenylases and promoting the degradation of its cognate targets (52–54, 58, 59). IFN-β expression is reported to be regulated at the posttranscriptional level (60–64), and its 3′ untranslated region (UTR) contains a perfect match to the TTP consensus binding site, UAUUUAU. To determine if TTP might regulate IFN-β production, we used mice with knock-in mutations in the endogenous TTP gene that change the two major MK2 phosphorylation sites, serines 52 and 178, to alanine. The mutant form of TTP cannot be phosphorylated and inactivated by MK2, and therefore, it functions as a constitutive destabilizer of its target mRNAs (51, 57). BMDMs from the TTP knock-in mice, as well as TTP knock-in/DUSP1 knockout double-mutant mice, were isolated, and LPS-induced IFN-β production was analyzed. As shown in Fig. 7, DUSP1 knockout macrophages produced more IFN-β mRNA than wild-type cells (Fig. 9C and D). TTP knock-in did not affect the initial induction of IFN-β mRNA in response to LPS (Fig. 9C). This finding held true for the TTP knock-in on both wild-type and DUSP1 knockout backgrounds (Fig. 9C). After 4 h of LPS stimulation, however, the TTP knock-in cells had lower levels of IFN-β mRNA than wild-type cells, consistent with destabilization of the IFN-β mRNA in the knock-in cells (Fig. 9D). Four hours after LPS stimulation, IFN-β mRNA levels in DUSP1 knockout BMDMs were higher than in wild-type cells. This increase in IFN-β mRNA was blunted in cells from TTP/DUSP1 double-mutant mice (Fig. 9D). As measured by actinomycin D chase experiments, 1 h after LPS stimulation, IFN-β mRNA was stable in wild-type and DUSP1 knockout cells; however, the TTP knock-in had a destabilizing effect on IFN-β mRNA (Fig. 9A). Four hours after the addition of LPS (Fig. 9B), DUSP1 deletion strongly stabilized IFN-β mRNA, and this effect was reversed by combining DUSP1 deletion with targeted mutation of TTP phosphoacceptor sites. Therefore, DUSP1 influenced expression of IFN-β via modulation of TTP phosphorylation, but this influence was more evident during the late phase of the response to LPS. Finally, the levels of IFN-β secreted into the media by the TTP knock-in mice was determined. In agreement with the results of the mRNA analysis following 4 h of LPS stimulation, TTP knock-in cells secreted less IFN-β than wild-type BMDMs. As shown in Fig. 7, DUSP1 knockout resulted in elevated IFN-β secretion, but this effect was reduced in DUSP1/TTP double-mutant cells (Fig. 9E).

## DISCUSSION

We show here that double knockout of MSK1 and -2 results in elevated induction of IFN-β mRNA levels relative to wild-type cells in response to LPS or poly(I·C). It also translated into increased secretion of IFN-β by MSK1/2 knockout macrophages relative to wild-type controls. Unexpectedly, this was not due to a direct effect of MSKs on the phosphorylation of transcription factors on the IFN-β promoter, nor was it due to the ability of MSKs to regulate IL-10 production. While double knockout of MSK1 and -2 can result in elevated TNF, IL-6, and IL-12 production in response to LPS, these effects are largely due to the reduced IL-10 production in MSK1/2 knockouts (21). In contrast, MSKs inhibit IFN-β production independently of IL-10 (Fig. 2 and 3). In the context of LPS-induced endotoxic shock, IFN-β knockout in mice has been found to be protective (9). Thus, the increase in IFN-β in MSK1/2 knockout mice may act in concert with the decrease in IL-10 production relative to wild-type animals to explain the previously reported sensitization of MSK1/2 knockout mice to LPS-induced endotoxic shock (21). The IFN-β promoter has been extensively studied, and a role for an enhanceosome consisting of IRF3, AP-1 (composed of ATF2 and c-jun), and NF-κB has been established in IFN-β expression in response to viral infection (38, 65). While in some circumstances, binding of NF-κB has been reported prior to activation, recruitment of c-jun/ATF2 and IRF3 to the IFN-β promoter requires stimulation (66). The importance of NF-κB to IFN-β induction may also be context and cell type dependent; for example, recent studies have found that knockout of the NF-κB subunit p50 or cRel reduced IFN-β induction in plasmacytoid, but not conventional, dendritic cells in response to viral infection (67). In mouse embryonic fibroblasts (MEFs), studies using p65/RelA knockout mice suggested that NF-κB was rate limiting for IFN transcription only under conditions of low IRF3 activation (68). Interestingly, IRF3 recruitment has been proposed to be more efficient when c-jun/ATF2 are already bound adjacent to the IRF binding site (66, 69).

**FIG 10:**
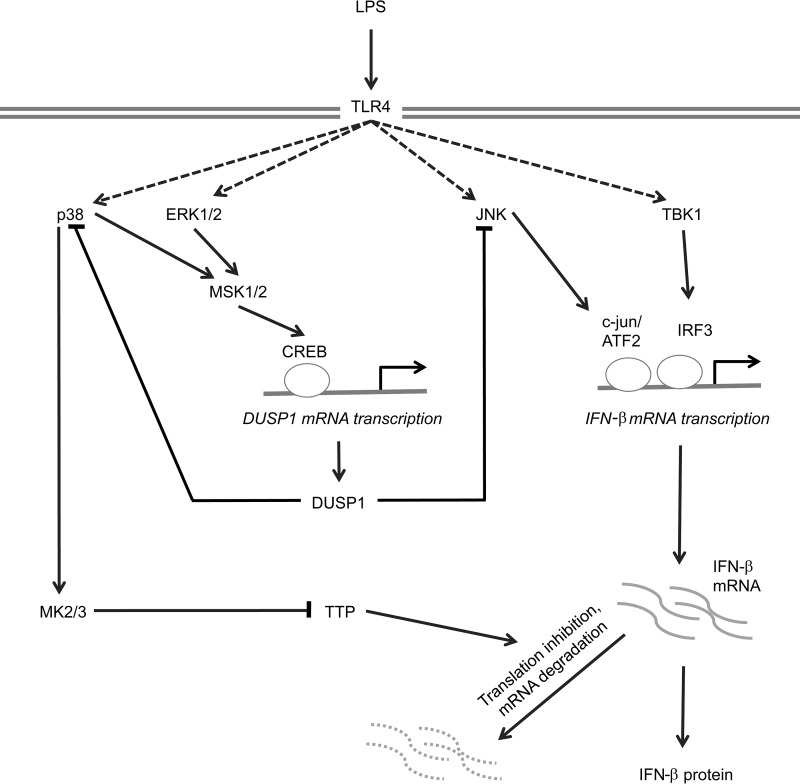
Proposed model of IFN-β transcriptional regulation downstream of MSKs. TLR4 agonists, such as LPS, stimulate the activation of the JNK/c-jun/ATF2 and Tbk1/IRF3 pathways to induce the transcriptional activation of the IFN-β promoter. TLR4 also activates the ERK1/2 and p38 MAPK pathways, resulting in the activation of MSK1 and -2 and the phosphorylation of the MSK substrate, CREB. CREB then helps induce transcription of the phosphatase DUSP1, upregulating DUSP1 protein levels. DUSP1 is then able to dephosphorylate p38 and JNK, resulting in reduced activation of these pathways. Loss of this MSK/CREB/DUSP1 pathway results in prolonged activation of JNK, c-jun, and ATF2, thus helping to drive IFN-β transcription. Prolonged activation of p38 also activates MK2, which phosphorylates TTP, thus preventing TTP from promoting IFN-β mRNA degradation.

Our data indicate that MSKs may regulate IFN-β transcription indirectly via the induction of the MAPK phosphatase DUSP1 (Fig. 10). MSKs regulated the induction of DUSP1 mRNA and protein in response to LPS, in part via the phosphorylation of CREB. MSKs have previously been shown to promote CREB-dependent transcription via its phosphorylation on Ser133 (28, 70), and binding sites for CREB have been identified in the DUSP1 promoter (71, 72). DUSP1 is also regulated posttranscriptionally, at the level of mRNA stability or translation (73–77). While our data do not exclude an effect of MSKs on DUSP1 mRNA stability, the decreased induction of the DUSP1 primary transcript in MSK1/2 knockout and CREB Ser133Ala knock-in cells suggests that MSKs act to control transcription from the DUSP1 promoter. The smaller effect of the CREB Ser133Ala knock-in than that of the MSK1/2 knockout on DUSP1 induction may reflect the ability of MSKs to phosphorylate ATF1 (28), a transcription factor closely related to CREB that binds to the same consensus sequence. We demonstrate here that the decrease in DUSP1 induction in response to LPS stimulation in MSK1/2-deficient cells coincides with an increase in the phosphorylation of JNK on its activation motif, a finding consistent with the reports of prolonged JNK activation in DUSP1 knockout macrophages (42–44). The increase in JNK activity in MSK1/2 knockout BMDMs in response to LPS results in an increase in the phosphorylation of c-jun, a transcription factor that forms part of the IFN-β enhanceosome. Of note, while many of the early studies on the IFN-β enhanceosome were performed in cell lines, siRNA knockdown of c-jun has been found to reduce LPS-stimulated IFN-β production in BMDMs (40). It is well established that phosphorylation of c-jun increases its activity (47, 48, 78, 79), suggesting that the increase in IFN-β mRNA in LPS-stimulated MSK-deficient BMDMs could result from the increase in JNK activity observed in these cells.

In both MSK1/2 and DUSP1 knockout macrophages, the increased expression of IFN-β mRNA was dependent on both JNK and p38 and was significantly impaired by combined inhibition of both pathways (Fig. 8A and B). The effect of JNK dysregulation might be explained by prolonged activation of c-jun and increased IFN-β transcription. However, the contribution of p38 to IFN-β overexpression appears to be independent of this mechanism, given that p38 inhibition did not affect c-jun phosphorylation.

Inflammatory responses of macrophages are constrained by the mRNA-destabilizing protein TTP. Mice lacking TTP spontaneously develop severe and pervasive inflammation that is largely mediated by overexpression of TNF, although several other inflammatory mediators are also dysregulated (80). Myeloid cell-specific TTP knockout does not cause the same pervasive syndrome but results in extreme sensitivity to lethal endotoxemia (81, 82). In macrophages, the MAPK p38 pathway regulates the expression of many inflammatory mediators at a posttranscriptional level via MK2-mediated phosphorylation and inactivation of TTP (54, 56, 59, 83). Substitution of the two key phosphoacceptor sites of endogenous TTP protein protected mice from endotoxemia by preventing MK2-mediated TTP inactivation (51). It was previously shown (57) that dysregulated p38 signaling in DUSP1 knockout macrophages caused overexpression of many inflammatory mediators by enhancing or prolonging p38 activation, promoting the phosphorylation and inactivation of TTP. Genes regulated by the DUSP1-p38-MK2-TTP axis were characteristically underexpressed by TTP knock-in macrophages and overexpressed by DUSP1 knockout macrophages but not by DUSP1 knockout macrophages in which TTP was also mutated to prevent its phosphorylation and inactivation.

We used a similar approach to test the hypothesis that DUSP1 controls the expression of IFN-β via modulation of TTP activity. In wild-type, TTP knock-in, DUSP1 knockout, and combined DUSP1 knockout–TTP knock-in macrophages, the expression of IFN-β mRNA and protein after 4 h of LPS treatment exactly conformed to the characteristic pattern described above (Fig. 9). Moreover, stabilization of IFN-β mRNA in DUSP1 knockout macrophages was dependent on TTP phosphorylation (Fig. 9D), demonstrating that IFN-β is posttranscriptionally regulated by DUSP1 and TTP in exactly the same manner as other inflammatory mediators, such as TNF and IL-1β (57). The IFN-β 3′ UTR contains a highly conserved ARE (63, 64), which has been shown to promote deadenylation of the IFN-β mRNA (63). Although TTP has not yet been directly implicated in regulating IFN-β expression, the ARE contains a perfect match to the consensus TTP binding site, UAUUUAU, and two additional, closely related sequences (62). Recent studies have used cross-linking and immunoprecipitation to identify transcriptome-wide targets of TTP (74, 75). Stringent analysis of both data sets using the TTP-Atlas website (http://ttp-atlas.univie.ac.at/) revealed only low-confidence peaks of binding of TTP in the IFN-β 3′ UTR. However, the LPS-induced expression of IFN-β mRNA was elevated by approximately 7-fold in TTP knockout macrophages (75), again suggesting that IFN-β is an authentic TTP target. In support of this conclusion, TTP knock-in macrophages underproduced IFN-β protein (Fig. 9E). The time-dependent modulation of IFN-β mRNA stability is also highly characteristic of TTP targets, in which rates of mRNA decay are tightly coupled to the activity of the p38 pathway (75, 81). Four hours after LPS treatment, when IFN-β mRNA stability was relatively low, the stabilizing effect of DUSP1 knockout was readily seen, and this effect was reversed by blocking TTP phosphorylation (Fig. 9B). However, the destabilizing effect of the TTP knock-in itself was not obvious. In contrast, 1 h after LPS treatment, when IFN-β mRNA was very stable in wild-type macrophages, the stabilizing effect of DUSP1 knockout could not be demonstrated, but decreased stability was very evident in TTP knock-in macrophages (Fig. 9A). Altogether, our data suggest that MSK1/2 and DUSP1 regulate the early phase of IFN-β expression principally at a transcriptional level, via JNK and likely via the phosphorylation of c-jun, and the late phase of IFN-β expression principally at a posttranscriptional level, via p38-dependent phosphorylation and inactivation of TTP.

DUSP1 is reported to be a target of TTP (73–77). We therefore considered the possibility that altering TTP function influences IFN-β expression indirectly by modulating MAPK signaling and the activation of phosphorylation-dependent transcription factors, such as c-jun. However, we were unable to detect differences of MAPK signaling in TTP knock-in macrophages (51). It therefore seems most likely that TTP regulates IFN-β expression directly at the posttranscriptional level, via the well-conserved ARE in its 3′ UTR.

The role of p38 in regulating IFN-β mRNA induction is therefore complex; while via MSK1 and -2 it can inhibit IFN-β mRNA induction due to increased DUSP1 expression, it can also, via MK2 and TTP, promote the stability and/or translation of IFN-β mRNA (Fig. 10). This potential for both positive and negative roles for p38 in IFN-β induction would result in context-dependent effects of p38 inhibition on IFN-β induction. This is borne out by reports in the literature; for example, in macrophages, p38 inhibitors are reported to reduce IFN-β induction in response to infection with Listeria monocytogenes, vesicular stomatitis virus, avian influenza virus H5N1, or Chlamydia muridarum but not induction by Sendai virus or poly(I·C) (84–87). JNK and p38 are not the only MAPKs to regulate IFN-β mRNA induction. The ERK1/2 pathway can negatively regulate IFN-β induction (88). In response to LPS, TPL2 is the MAP3K required for ERK1/2. Knockout of TPL2 resulted in the inhibition of ERK1/2 activation but increased transcription and secretion of IFN-β (88), as well as increased susceptibility to infection with the intracellular bacteria Mycobacterium tuberculosis and L. monocytogenes (89). In line with this, knockout of p105, which is required to maintain Tpl2 protein levels, also increased IFN-β mRNA induction (90).

Taken together, our data illustrate complex regulation of IFN-β mRNA induction in macrophages that involves, in addition to the widely studied TBK/IRF3 pathway, regulation via the p38/MK2/TTP and JNK/c-jun pathways in response to TLR4 activation.

## MATERIALS AND METHODS

### Animals.

MSK1/2 knockout, CREB Ser133Ala knock-in, TTP S52A/S178A double-knock-in, and DUSP1 knockout mice have been described previously (28, 51, 91, 92). All experiments were carried out on mice that had been backcrossed onto C57BL/6J mice (Charles River Laboratories) for a minimum of 12 generations. The mice were given free access to food and water and maintained in individually ventilated cages under specific-pathogen-free conditions. The work was carried out in accordance with European Union and UK regulations. All procedures were subject to local ethical review and were carried out under a UK project license.

### *In vivo* LPS injection.

Mice 6 to 8 weeks of age were injected i.p. with 1.8 mg/kg of body weight LPS from Escherichia coli strain O26:B6 (Sigma; L2654) dissolved in phosphate-buffered saline (PBS). The mice were culled via exposure to a rising concentration of CO_2_, followed by confirmation that death had occurred by cervical dislocation 1 h or 4 h after LPS injection, and plasma samples were collected for cytokine analysis.

### Cell culture.

BMDMs were cultured as described previously (93). Briefly, bone marrow was flushed from the femurs of one mouse using PBS. Cells were then pelleted by centrifugation and cultured on bacterial-grade plastic for 7 days in BMDM medium (Dulbecco's modified Eagle's medium [DMEM] supplemented with 10% heat-inactivated fetal bovine serum, 2 mM l-glutamine, 100 U/ml penicillin G, 100 μg/ml streptomycin, 0.25 μg/ml amphotericin, and 5 ng/ml recombinant macrophage colony-stimulating factor [M-CSF]). The cells were then detached by scraping in EDTA (Invitrogen) and replated on tissue culture plastic in BMDM medium. The cells were stimulated with either 100 ng/ml LPS (Sigma) or 10 μg/ml poly(I·C) (Invivogen). Where indicated, cells were preincubated for 1 h with 2 μM PD184352 or 1 μM VX745 or for 3 h with 3 μM JNK-In-8. The selectivities of the inhibitors have been reported previously (50, 93, 94).

### mRNA expression analysis.

Total RNA was isolated using microRNeasy kits (Qiagen) and reverse transcribed using iScript (Bio-Rad). mRNA levels were determined by quantitative PCR (qPCR) using SYBR green-based detection. 18S or GAPDH (glyceraldehyde-3-phosphate dehydrogenase) mRNA levels were used as normalization controls. Primers for TNF and DUSP1 (21) and IFN-β (95) have been described previously. For detection of the DUSP1 primary transcript, the primer sequences ATGACTGCAAGAGGCAGACC and GGCCTGGCAATGAACAAACA were used.

### Cytokine analysis.

IL-10 was measured using a Luminex-based assay (Bio-plex; Bio-Rad) according to the manufacturer's protocol. IFN-β was measured using a VeriKine enzyme-linked immunosorbent assay (ELISA) (PBL Interferon Source) or via a Luminex-based assay (eBioscience), as indicated in the figure legends.

### Immunoblotting.

For immunoblotting, cells were lysed in 50 mM Tris-HCl (pH 7.5), 1 mM EGTA, 1 mM EDTA, 1 mM sodium orthovanadate, 50 mM sodium fluoride, 1 mM sodium pyrophosphate, 10 mM sodium glycerophosphate, 0.27 M sucrose, 1% (vol/vol) Triton X-100, 0.1% (vol/vol) 2-mercaptoethanol, and complete proteinase inhibitor cocktail (Roche). Lysates were clarified by centrifugation (13,000 rpm for 10 min at 4°C), and the supernatants were snap-frozen and stored at −80°C. The protein concentration was determined with Coomassie protein assay reagent (Thermo Scientific). Proteins were separated on 10% polyacrylamide gels, and immunoblotting was carried out using standard techniques. Antibodies against phospho-Ser172 TBK1, total TBK1, phospho-Thr180/Tyr182 p38, total p38, phospho-Thr202/Tyr204 ERK, total ERK, phospho-Ser133 CREB, total JNK, phospho-Ser63 c-jun, total c-jun, and phospho-Thr334 MK2 were from Cell Signaling. The antibody against phospho-Thr183/Tyr185 JNK was from Invitrogen, the antibody against DUSP1 was from Santa Cruz Biotechnology (sc-373841), and the antibody raised against total MSK1 was described previously (28). Secondary antibodies were conjugated to horseradish peroxidase, and bands were visualized via Clarity ECL (Bio-Rad) using either film or a Li-Cor Odyssey Fc scanner. Quantification was performed using Image Studio Lite software driving the Li-Cor Odyssey Fc scanner.
